# Supplementary material for: Hypoxia ameliorates intestinal inflammation through NLRP3/mTOR downregulation and autophagy activation
Source: Nat Commun. 2017 Jul 24;8:98. doi: 10.1038/s41467-017-00213-3 (PMC5524634; doi:10.1038/s41467-017-00213-3)
Supplement: Supplementary file 1 — Supplementary Information [file 41467_2017_213_MOESM1_ESM.pdf]

File name: Supplementary Information
Description: Supplementary figures.

File name: Peer review file
Description:

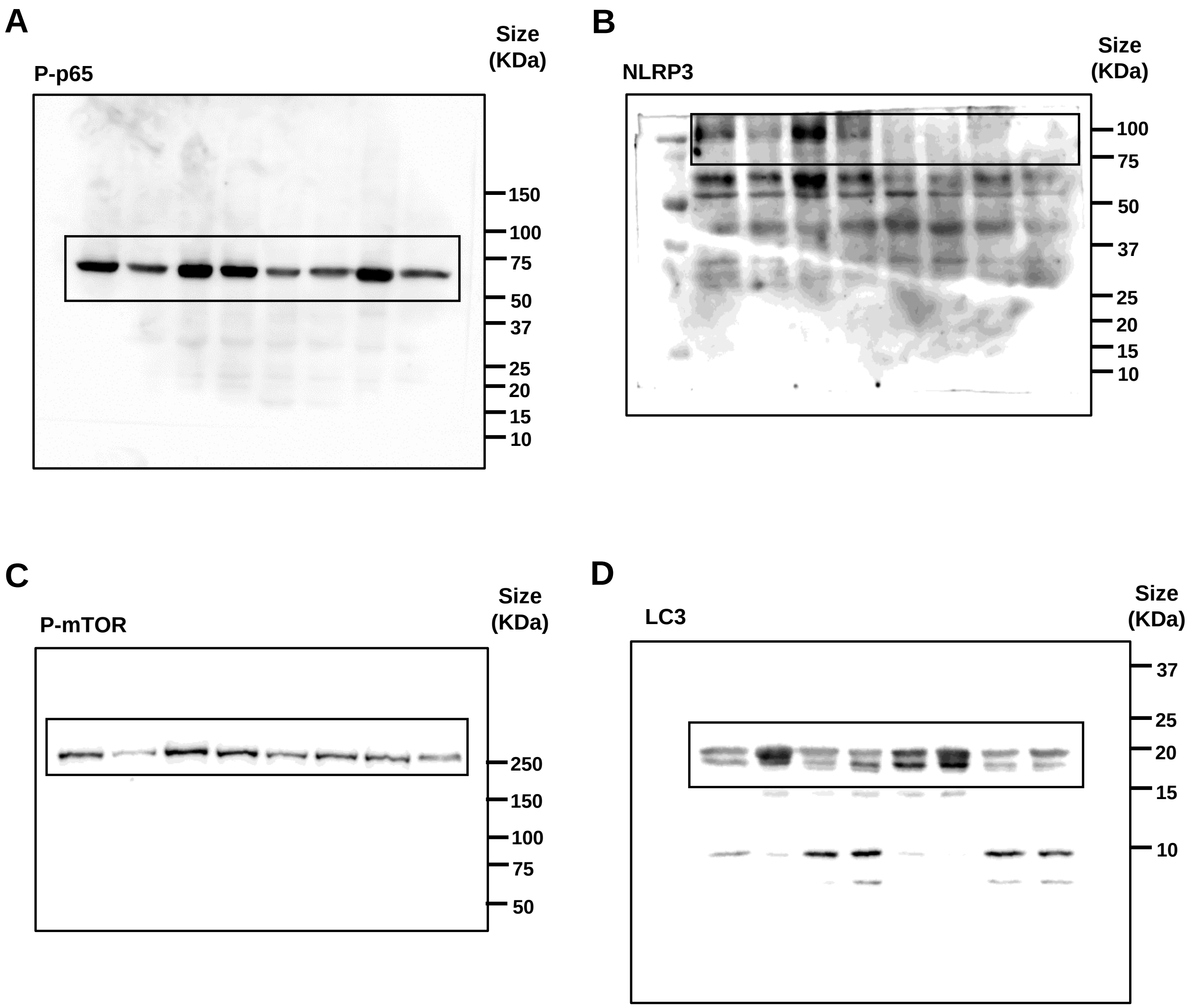

Supplementary Figure 1. Uncropped versions of blots shown in Figure 2H

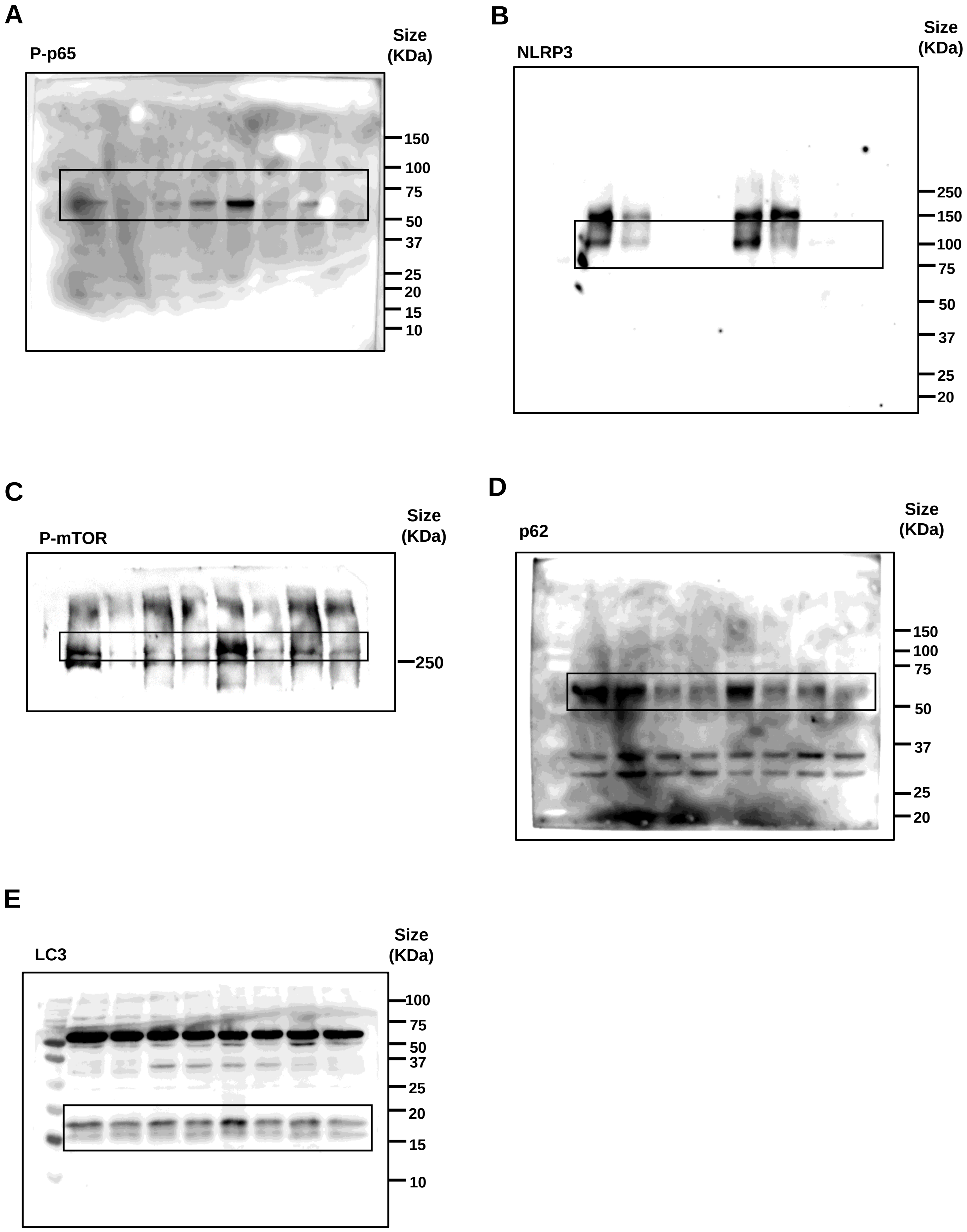

Supplementary Figure 2. Uncropped versions of blots shown in Figure 3H

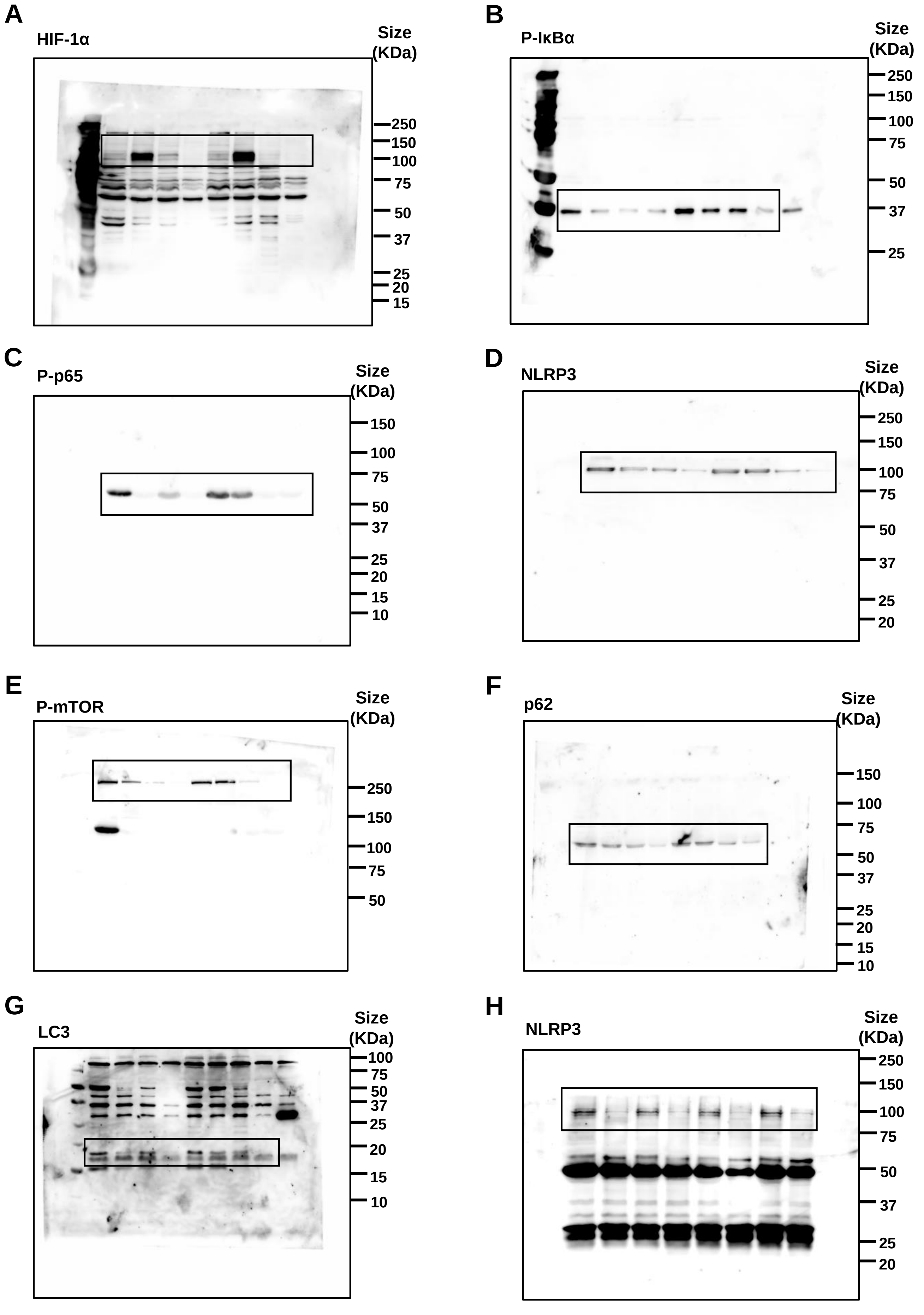

Supplementary Figure 3. Uncropped versions of blots shown in Figure 4A (A-G) and Figure 4E (H)

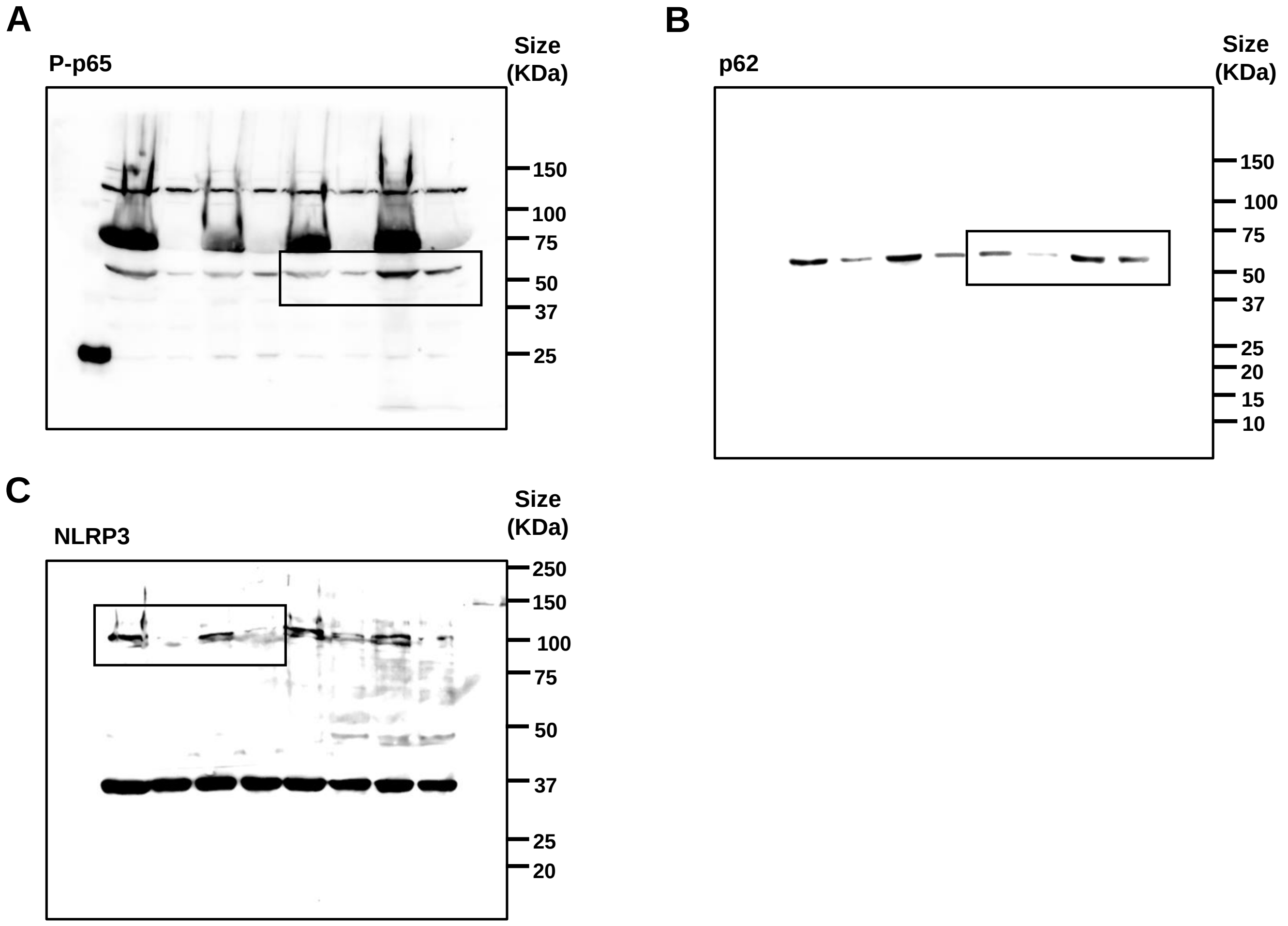

Supplementary Figure 4. Uncropped versions of blots shown in Figure 5C

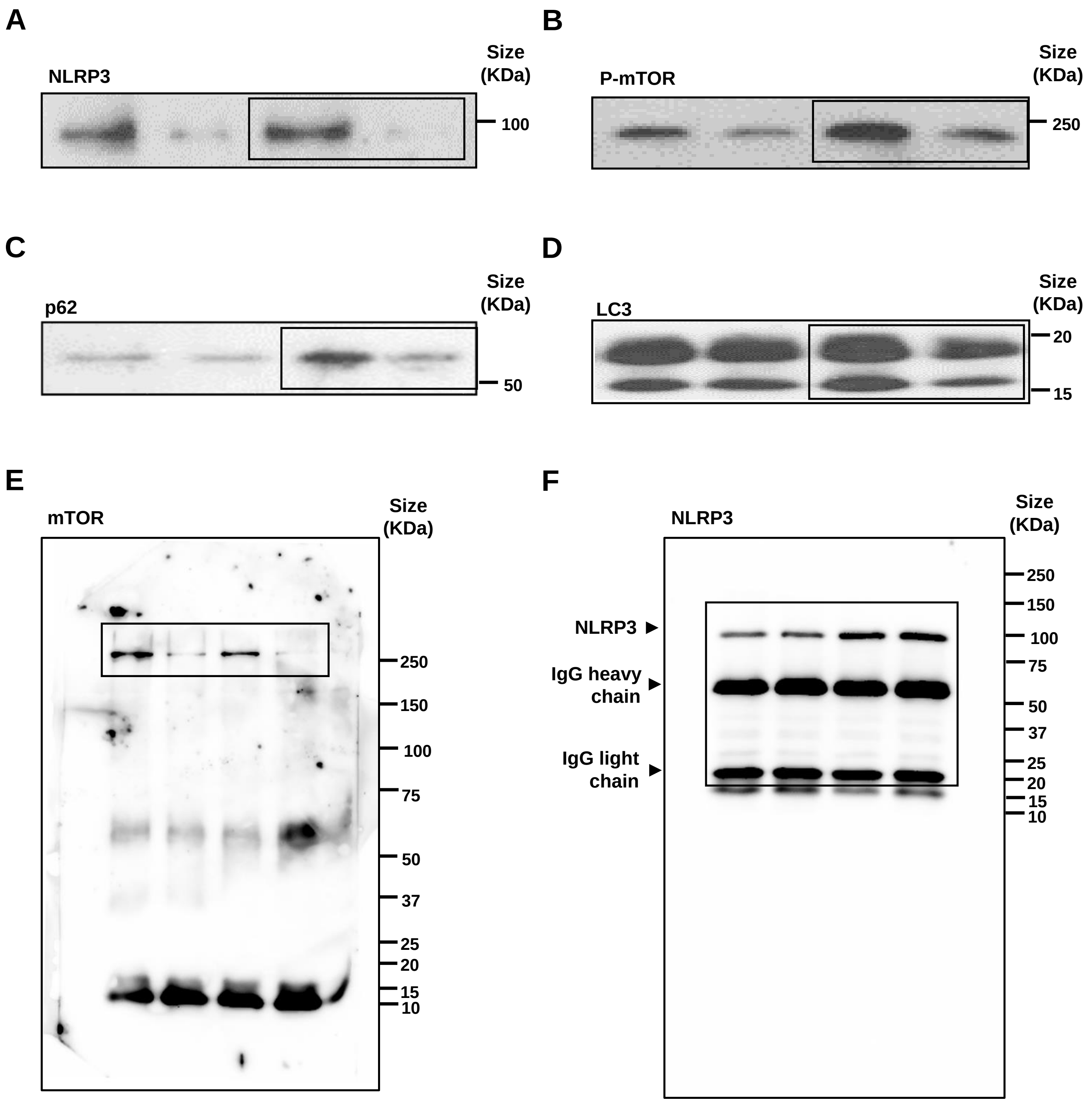

Supplementary Figure 5. Uncropped versions of blots shown in Figure 6A (A-D) and B (E, F)

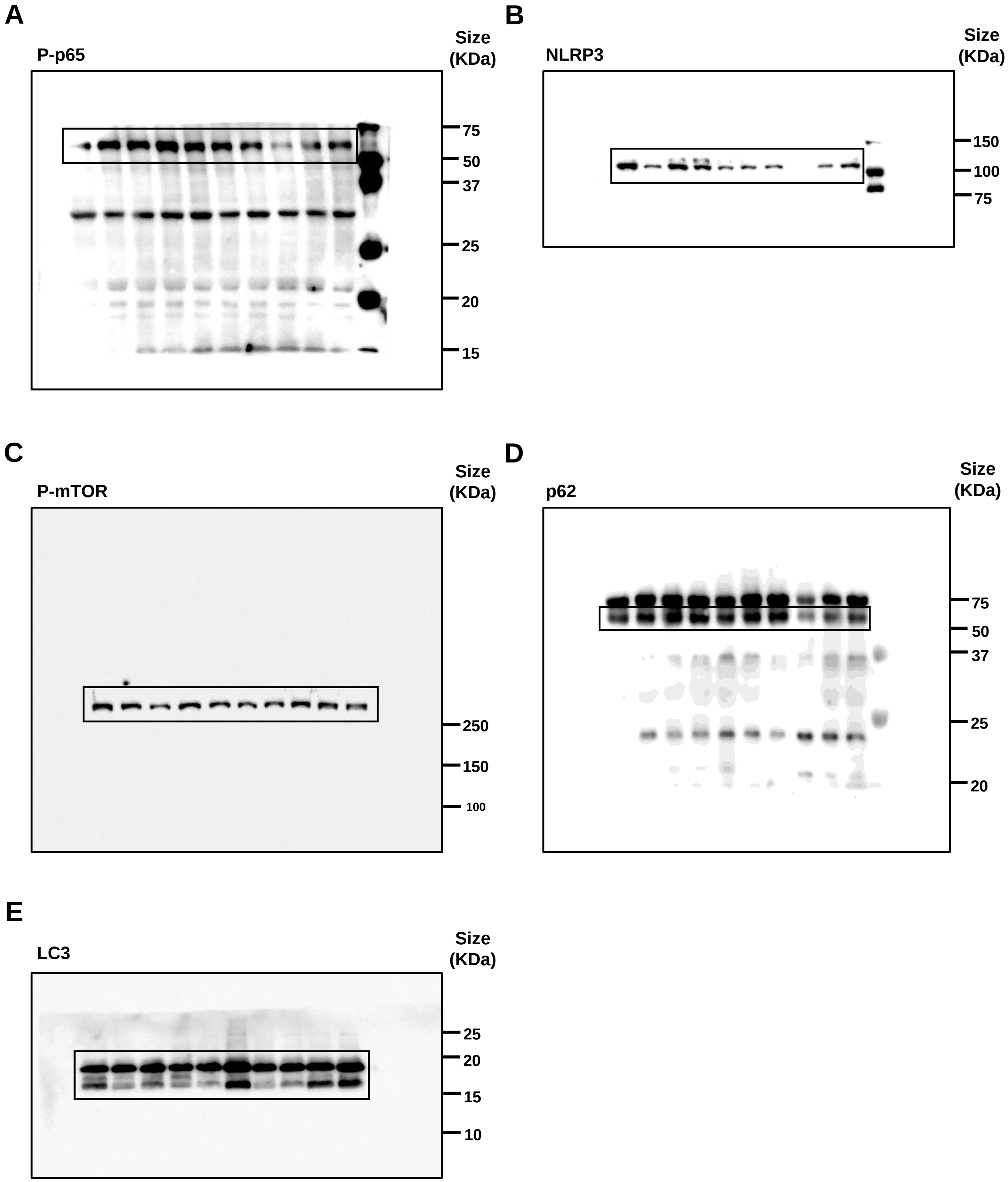

Supplementary Figure 6. Uncropped versions of blots shown in Figure 7N
